# Natural products from the termite *Nasutitermes corniger* lowers aminoglycoside minimum inhibitory concentrations

**DOI:** 10.4103/0973-1296.59958

**Published:** 2010-02-13

**Authors:** Henrique D. M. Coutinho, Alexandre Vasconcellos, Hilzeth L. Freire-Pessôa, Carlos A. Gadelha, Tatiane S. Gadelha, Geraldo G. Almeida-Filho

**Affiliations:** *Laboratório de Pesquisa em Produtos Naturais, Departamento de Ciências Biológicas, Universidade Regional do Cariri, Crato (CE), 63105-000, Brazil*; 1 *Universidade Federal do Rio Grande do Norte, Departamento de Botânica, Ecologia e Zoologia, Natal (RN). 59072-900, Brazil*; 2 *Universidade Federal da Paraíba-UFPB, Departamento de Biologia Molecular-DBM, 58051-900, Brazil*

**Keywords:** Aminoglycosides, antibiotic modifying activity, ethnozoology, isopteran, *N. Corniger*, termite, zootherapy

## Abstract

Bacterial infectious agents present a risk to populations, as they are responsible for high morbidity and mortality. For combating these pathogens, our main line of defense is the use of antibiotics. However, indiscriminate use of these drugs develops resistant strains to these same drugs. The present study has tested the antibacterial and modifying antibiotic activity of natural products from *Nasutitermes corniger* (Termitidae) (Motschulsky), a termite used in folk medicine in Northeast Brazil, by the microdilution and checkerboard methods, respectively. In this study, the aqueous extract from the nest of *N. corniger* (ANCE) was prepared and tested with chlorpromazine (CPZ) for its antimicrobial activity, using the microdilution method. CPZ and ANCE were used independently and also in combination with aminoglycosides, against a strain of *Escherichia coli* resistant to these antibiotics, to determine the participation of efflux systems in resistance mechanisms. The fractional inhibitory concentration (FIC) index was calculated and evaluated for the occurrence of synergism, using the checkerboard method. The minimum inhibitory concentrations (MIC) and minimum bactericidal concentrations (MBC) values were ≥ 2048 μg/mL for both strains of *E. coli* assayed, indicating low antibacterial activity. However, synergism was observed with kanamycin when the decoction was used, but when chlorpromazine was used, synergism was observed with kanamycin, amikacin, and neomycin. This synergism with CPZ indicated the involvement of an efflux system in the resistance to these aminoglycosides. Therefore, it was suggested that the natural products from *N. corniger* could be used as a source of zoo-derived natural products with kanamycin-modifying activity, resulting in a new approach against bacterial resistance to antibiotics.

## INTRODUCTION

Although plants are more commonly studied around the world for their medicinal potential, animals or animal parts have also been widely used in Brazilian traditional medicine and have played a significant role in healing practices.[1–3] Products from several species of Insecta have been used as remedies.[1,2,4–6] Among these species is the Neotropical termite *Nasutitermes corniger* (Termitidae) (Motschulsky), which is commonly used in traditional medicine in Northeast Brazil. *N*. *corniger* is distributed from southern Mexico to northern Argentina and the West Indies,[7–11] inhabiting both semi-arid and tropical rainforest ecosystems. In South America, this species is highly adaptable to the colonization of contrasting habitats in urban, agricultural, and natural environments.[12,13] *N*. *corniger* builds arboreal carton nests with a population that can exceed 400,000 individuals/nest[14] and a density that ranges from 22.1 to 47.1 nest/ha in tropical rainforests.[12,15] Scheffrahn *et al.*,[11] based on the morphological, genetic, and biogeographic lines of evidence have made *N*. *costalis* (Termitidae)(Holmgren) a synonymous with *N*. *corniger*. According to Constantino,[10] the congeneric species *N*. *araujoi* (Termitidae)(Roonwal and Rathore), *N*. *globiceps* (Termitidae)(Holmgren), and *N*. *tatarendae* (Termitidae)(Holmgren) can also be synonymous with *N*. *corniger*.

With the increase in resistance to antibiotics, natural products represent an interesting alternative.[16,17] Many products have been evaluated not only for direct antimicrobial activity, but also as resistance-modifying agents.[18,19] Several chemical compounds from synthetic or natural sources, such as, phenothiazines and natural products, have direct activity against many species of bacteria, enhancing the activity of specific antibiotics, reversing the natural resistance of specific bacteria to several antibiotics, and promoting the elimination of plasmids from bacteria such as *Escherichia coli*. Inhibition of plasma membrane-based efflux pumps has been observed as well.[20,21] The enhancement of antibiotic activity or the reversal of antibiotic resistance by natural or synthetic non-conventional antibiotics results in the classification of these compounds as modifiers of antibiotic activity. Aminoglycosides are potent bactericidal antibiotics targeting the bacterial ribosome, but the increase in bacterial resistance to aminoglycosides is widely recognized as a serious health threat.[20] In *E. coli,* the main mechanisms of resistance to aminoglycosides are their active efflux and enzymatic inactivation.[21] The present study has evaluated a decoction of *N*. *corniger* nests and CPZ as a resistance-modifying agent in a strain of *E*. *coli* resistant to aminoglycosides.

## MATERIALS AND METHODS

### Strains

The strain used was a clinical isolate of *Escherichia coli* (EC27), resistant to neomycin and gentamicin (low level) and to amikacin and kanamycin. The *Escherichia coli* strain EC - ATCC8539 was used as a positive control sensitive to aminoglycosides. All strains were maintained on heart infusion agar slants (HIA, Difco), and prior to assay, the cells were grown overnight at 37°C in a brain heart infusion (BHI, Difco).

### Zoological material

*Nasutitermes corniger* was collected in the county of Alagoa Nova, Paraíba, Brazil (21°58′N, 89°36′W) during June 2007. The samples were authenticated by Dr. Alexandre Vasconcellos at the Botany, Ecology, and Zoology Department, UFRN. Voucher specimens (CICB 68 and CICB 69) were deposited in the Isoptera Collection of the Bioscience Center, Universidade Federal do Rio Grande do Norte-UFRN.

### Preparation of aqueous extract of *N. corniger* nest (ANCE)

An amount of 200 g of termite nest was collected and powdered. The powdered material was extracted by maceration using 100 mL of sterile water as a solvent, at room temperature. The extract was allowed to stand for 72 h at room temperature. The aqueous extract was filtered and assayed to determine antibacterial activity.

### Drugs

Chlorpromazine, gentamicin, kanamycin, amikacin, and neomycin were obtained from SIGMA, St. Louis, USA. All the drugs were dissolved in sterile water.

### Drug susceptibility test and determination of fractional inhibitory concentration

The minimum inhibitory concentrations (MICs) of ANCE, antibiotics, and CPZ were determined in BHI by the microdilution method, using suspensions of 10^5^ CFU/mL and a drug concentration range of 1024 to 1 μg/mL (two-fold serial dilutions).[22] MIC was defined as having the lowest concentration at which no growth was observed. For the evaluation of ANCE as a modulator of antibiotic resistance, the MICs of the antibiotics were determined in the presence of ANCE and CPZ at a sub-inhibitory concentration and the FIC was calculated. CPZ being an inhibitor of the efflux pump was used in this study to demonstrate the presence or absence of resistance by this mechanism and to verify if it was affected by ANCE. The fractional inhibitory concentration (FIC) was used to interpret the tube dilution method results and calculated as follows:[23] FIC of drug A = MIC of drug A in combination with ANCE or CPZ/MIC drug A alone. Synergy was defined as an FIC ≤ 0.5, indifference was defined as 4 ≥ FIC > 0.5, and antagonism was defined as an FIC >4. The plates were incubated for 24 h at 37°C. CPZ was used as the positive control for efflux pump inhibition.

## RESULTS AND DISCUSSION

ANCE did not show substantial antibacterial activity at 1024 μg/mL against the strains assayed (MIC for both μ 2048 μg/mL). However, when ANCE was added to the growth medium at 256 μg/mL, a reduction of the MIC for kanamycin was observed in the *E*. *coli* 27 strain (but not with ATCC 8539), demonstrating a synergistic effect of this natural product with this aminoglycoside [Table 1].

**Table 1:** Evaluation of the modifying antibiotic activity of the decoct from the nest of *N. Corniger* (256 μ/ml) and CPZ (16 μ/ml) against aminoglycosides

Antibiotics	EC^a^ 27			EC ATCC8539		
		
	MIC	MIC combined		MIC	MIC combined	
				
	-	ANCE^b^/FIC^c^	CPZ^a^/FIC	-	ANCE/FIC	CPZ/FIC
Gentamicin	8	8/1 (I)	8/1 (I^f^	8	8/1 (I)	8/1 (I)
Kanamycin	64	32/0,5 (S^e^)	8/0,12 (S)	1024	1024/1 (I)	1024/1 (I)
Amikacin	32	32/1 (I)	16/0,5 (S)	8	16/2 (I)	16/2 (I)
Neomycin	64	64/1 (I)	8/0,12 (S)	64	128/2 (I)	128/2 (I)
CPZ	64	-	-	512	-	
ANCE	≥2048	-	-	≥2048	-	-

a EC - *Escherichia coli*

b ANCE - Aqueous extract of *N. corniger* nest

c FIC - Fractional inhibitory concentration

^d^CPZ - Chlorpromazine

e S - Synergism

f I - Indifferent

Synergism between CPZ and gentamicin was not observed, but CPZ did show synergism with the other aminoglycosides, which is suggestive of the occurrence of efflux pumps against the latter. As the same effect was observed by using ANCE or CPZ, it is possible that the decoction affected the efflux resistance mechanism [Table 1].

Evidence of the antimicrobial activity of products isolated from termites has been reported. Peptides such as, spinigerin and termicin, isolated from *Pseudocanthotermes spiniger* (Termitidae) (Sjostedt), showed antifungal and antibacterial activities.[24,25] Bioinformatics and molecular biology studies on the Australian termites of the genus *Nasutitermes* demonstrated their potential as producers of antimicrobial peptides,[26,27] but as far as we know, there have been no previous reports on the antimicrobial activity of natural products from *N*. *corniger* or synergism between products of any genus of termites with aminoglycosides or any other antibiotic.

Phenothiazines, such as CPZ, probably act on the plasma membrane of the bacteria, affecting their efflux pumps.[28–30] This permeability modification could enhance the activity of antibiotics that act within the cell, such as the aminoglycosides. Efflux pumps as resistance mechanisms of *E*. *coli* have been known since the 1980s;[31] they belong to the RND (resistance nodulation division) family and represent a mechanism of multidrug resistance (MDR), having the antibiotic resistance to agents such as aminoglycosides.[32]

Animals have been methodically tested by pharmaceutical companies as sources of drugs for modern medical science[33] and the current percentage of animal sources for producing essential medicines is quite significant. The chemical constituents and pharmacological actions of some animal products are already known to some extent and ethnopharmacological studies focused on animal medicines may be very important in order to clarify the eventual therapeutic usefulness of this class of biological remedies.[34] As pointed by Alves and Rosa,[1] further ethnopharmacological studies are necessary to increase our understanding of the links between traditional uses of faunistic resources and conservation biology, public health policies, sustainable management of natural resources, and biological prospecting.

The results obtained indicate that the decoction of the nest of *N*. *corniger* (and possibly of other termites) could be a source of natural products capable of modifying antibiotic activity to be used against multidrug-resistant bacteria. These agents when used as an adjuvant in antibiotic therapy could make it possible to use lower antibiotic doses with an effective outcome, but without the risk of pathogenic microorganisms developing resistance. Thus, new investigations of these agents isolated from animals and plants could lead to interesting tools against multidrug resistance in bacteria.
